# Beta-Thalassemia in Iran: New Insight into the Role of Genetic Admixture and Migration

**DOI:** 10.1100/2012/635183

**Published:** 2012-12-18

**Authors:** Ali Reza Rezaee, Mohammad Mehdi Banoei, Elham Khalili, Massoud Houshmand

**Affiliations:** ^1^Department of Animal Science, Faculty of Agriculture, Ferdowsi University of Mashhad, Mashhad, Iran; ^2^Department of Medical Genetics, National Institute of Genetic Engineering and Biotechnology, Tehran 14155-6343, Iran; ^3^Department of Genetics, Special Medical Center, Tehran, Iran

## Abstract

Iran with an area of 1.648 million km^2^ is located between the Caspian Sea and the Persian Gulf. The Iranian population consists of multiethnic groups that have been influenced by various invasions and migration throughout history. Studies have revealed the presence of more than 47 different **β**-globin gene mutations responsible for **β**-Thalassemia in Iran. This paper is an attempt to study the origin of **β**-Thalassemia mutations in different parts of Iran. Distribution of **β**-Thalassemia mutations in Iran shows different patterns in different areas. **β**-Thalassemia mutations have been a reflection of people and area in correlation with migration and origin of ancestors. We compared the frequencies of **β**-globin mutations in different regions of Iran with those derived from neighboring countries. The analysis provided evidence of complementary information about the genetic admixture and migration of some mutations, as well as the remarkable genetic classification of the Iranian people and ethnic groups.

## 1. Introduction

The Iranian population consists of several ethnic groups most of whom are of “Aryan origin.” The strategical position of Iran as a crossroad between the west and the eastern civilizations has impressed many to determine the circumstances of gene flow. Thalassemia is the commonest monogenic disorder in the world and globally it is estimated that there are 270 million carriers, of which 80 million are carriers of *β*-Thalassemia with about 60000 affected children born annually [1]. More than 200 mutations affecting the *β*-globin gene are now known to result in a phenotype of *β*-Thalassemia. It affects different ethnic and geographical groups with miscellaneous prevalence in different regions [2, 3]. Association studies for screening the distribution patterns of *β*-Thalassemia mutations may have a source of information in population genetics science and could be tools as molecular tags in the heredity of human populations. Any geographic distribution of specific mutations may represent information on the place of origin of the genetic change that generated it. Therefore, we are able to analyze specific genetic links between different populations. Since the knowledge of the mutation spectrum present in a population is essential for the molecular diagnosis and prevention of hemoglobinpathies, the knowledge of the ethnical background of a particular area is equally important. Our study provides information on the history and origin of the different *β*-Thalassemia mutations, population movements, and the relationship between the different affected groups in the country. To improve the construction of a geographical map, results from neighboring countries have also been compared. This paper is based on the reports from the literature together with previously unpublished data collected from the diagnostic service in Iran. Author's efforts have been based on drawing data out from all local, international references, and presented abstracts in national or international congresses. All data (published and unpublished) are classified regarding to different regions and ethnicities and then added to other data (Table 1).

## 2. Materials and Methods

All materials and methods were used from 150 *β*-Thalassemia patients from Medical Genetic Laboratory (Special Medical Center, Tehran) and other publication from Iranian *β*-Thalassemia patients (Akbari, *n* = 45; Drakhshandeh, *n* = 96; Najmabadi, *n* = 164) [4–6].

## 3. Thalassemia in Iran

**Figure 1:**
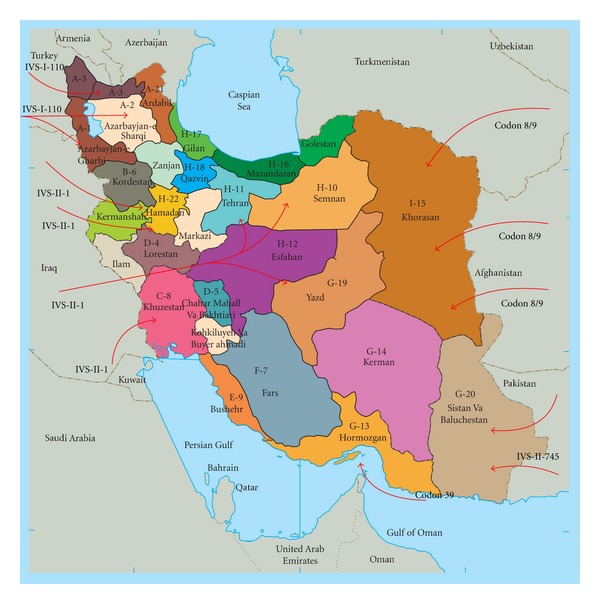
Distribution pattern of different mutations of *β*-Thalassemia in 21 provinces of Iran.

Among the eastern Mediterranean region, Iran is one of the major centers for the prevalence of *β*-Thalassemia. Regarding to high consanguinity among population, it is estimated that there are between two and three million *β*-Thalassemia carriers and 25,000 patients in Iran. Like many other countries in the region, a large number of Thalassemia patients are *β*-Thalassemia [7]. Alpha Thalassemia is not as prevalent as *β*-Thalassemia in Iran [8]. The gene frequency of *β*-Thalassemia, however, is high and varies considerably between areas. Recent studies have revealed the presence of more than 47 different *β*-globin gene mutations responsible for *β*-Thalassemia in Iran. The predominant IVS-II-1 (G > A; beta0) mutation is followed by decreasing order of frequency by the IVS-I-5 (G > C, beta+), the codons 8/9 (+G, beta0), the IVS-I-110 (G > A, beta+), the IVS-I-1 (G > A, beta0), 25 bp deletion (beta0), the IVS-I-6 (T > C, beta+), the codon 5 (-CT, beta0), and codon 39 (C > T, beta0) mutations. This group of mutations covers more than 85% of the total *β*-Thalassemia defects present in Iran [6, 9]. We compared *β*-Thalassemia mutations based on geographical area and frequency of mutation. Distribution of *β*-Thalassemia mutations in Iran shows different pattern in different area. In general, *β*-Thalassemia mutations have been a reflection of people and area in correlation with migration and origin of ancestors. We evaluated nine regions (A, B, C,…, I) which cover all of throughout Iran. Molecular screening revealed distribution pattern of different mutations of *β*-Thalassemia in 21 provinces (Figure 1, Table 1).

**Region A**

This region defines northwest of Iran which includes three provinces with common ethnicity, that of the Azeri ethnic group, the IVS-II-1 (G > A), codons 8/9 (+G), IVS-I-110 (G > A), and IVS-I-1 (G > A) mutations are most frequent in this area. Distribution pattern among these three provinces shows differences due to geographical region. IVS-II-1 (G > A) is a frequent mutation in the two northwestern provinces (West and East Azerbaijan provinces), region A-1 and A-2, respectively (Figure 1) [10–12]. There are different patterns of the prevalence of *β*-globin among the Azeri ethnic group, so that in the north of two provinces (region A-3, Figure 1) codons 8/9 (+G) are the most frequent mutation which has similarity with those in the Northeast of Iran. It is thought that the codons 8/9 (+G) was transferred by the big migration of the Seljuk Turks and the invasion by the Mongols to the north in 1219. Also, we observed significant differences between East Azerbaijan and West Azerbaijan provinces, respectively. Samplings from the latter province included regions with dominant Kurdish ethnicity which showed that mutations are similar to the Khug region in Kurdistan of Iraq [13]. The pattern in this region is more or less the same as in the Mediterranean regions [10]. IVS-I-110 (G > A) as an East Mediterranean mutation reaches a high frequency in some countries of the Arabian Peninsula, a fact that may be explained by gene flow and founder effect [14]. It decreases from the Northwest to the East and Center and other parts of Iran. Ottoman Empire role in admixture of cultural and social character of areas under their control is apparent. Primary documentation of the empire's relations with other powers is to be found in the archives of thirty-nine nations.

**Region B**

IVS-II-1 (G > A), codons 8/9 (+G), IVS-I-1 (G > A), and codon 8 (−AA) are the most frequent of the *β*-globin mutations in areas of Kurdistan [15]. It has been reported that codons 36/37 (−T) have a Kurdish origin that is prevalent in Kurdistan region, an area residing between Iran, Iraq and Turkey. Our study showed a low frequency of codons 36/37 (−T) in comparison to other areas. codon 8 (−AA) is another relative frequent mutation in the *β*-globin gene among populations who settle in the Northwest and west. Association studies showed this mutation has been observed frequently in the eastern parts of Turkey and Azerbaijan. Codon 8 (−AA) is the most frequent mutation of the *β*-globin gene in Azerbaijan [16]. The last country separated from Iran during 150 to 200 years ago. Contrary to previous studies, present investigation showed that this mutation is very low in the northwest among the Azeri ethnic group of Iran, so we could suppose a different origin for the two ethnic groups with a common name, or absence of any movement of codon 8 (−AA) toward this region. The first could be true especially due to dissimilarity in the most frequent *β*-globin mutations between the two regions regard to the evident migration from Azerbaijan to Iran during past century. On the other hand, IVS-II-1 (G > A) with frequency of 31.8% was similar to pervious studies and surrounding regions, also codon 44 (−C) showed frequency of 4.55% among Kurd ethnic group, while Rund et al. reported it as a frequent mutation (31.2%) in the Jews of Kurdistan in north of Iraq [17]. This study showed relative frequency for codon 44 (−C) (4.5%–9.3%) in northwest and West of Iran.

**Region C**

The area in southwest is near the Arabian Peninsula, IVS-I-1 (G > A), codons 8/9 (+G), IVS-I-110 (G > A), IVS-II-1 (G > A), IVS-I-6 (T > C) and IVS-I-5 (G > C) are frequent mutations that cause 86% of *β*-Thalassemia. Frequency of all mutations had approximate similarities that showed the population gained mutations from different origins. Land fertility has prepared suitable circumstances for survival for long periods. In fact, many ethnicities have migrated or passed into or through this ancient territory during the past 3000 to 5000 years old [10]. Following the advent of Islam, small and numerically inferior Arab tribes migrated to inland Iran. This migration caused the introduction of mutations such as IVS-I-6 (T > C) [18, 19] which has a frequency as high as 11% in this area that disappeared in other areas with the exception of the northwest where the IVS-I-6 (T > C) could have been introduced through Turkey [20].

IVS-I-1 (G > A) is the most frequent mutation in southwest. IVS-I-1 (G > A) has been described initially as a Mediterranean mutation. This mutation is at high frequency in east of the Mediterranean countries such as Lebanon, Palestine, and Syria [21]. It has been suggested that the Ottoman Empire played an important role in the migration of the IVS-I-1 (G > A) mutation from these countries to Iran during Ottoman rules.

**Region D**

As it is clear in Figure 1, this region is in the west of central of Iran, and this area belongs to two ethnic of Iranian group, called the Lur and the Bakhtiaris shown as region D-4 and D-5 in Figure 1, respectively. Codons 36/37 (−T) are the most frequent mutation within these groups (31% to 34%) [22]. It could be supposed that the origin of codons 36/37 (−T) is related to the above mentioned area not observed in other areas inside or outside such as near neighboring Turkey [23].

**Region E**

This area around the Persian Gulf has shown a different pattern, so that the 25 bp deletion is a frequent mutation in this region and region F [24–26]. On the other hand, the 25 bp deletion is most prevalent in Saudi Arabia and Bahrain showing frequencies of 14% and 36%, respectively. Due to frequency of 25 bp deletion within Iran and Bahrain and other regions of the Arabian peninsula as old parts of Iran in the past centuries [14, 27], It is said the origin of this mutation is Asian-Indian as well as, it has been proposed that there is another origin for 25 bp deletion in south of Iran littoral of Persian Gulf.

**Region F**

This region is in the south and has the following mutations of the *β*-globin: IVS-II-1 (G > A), 25 bp deletion and codon 8 (−AA), in frequencies of 47.4%, 21.6% and 8.6%, respectively. Overall, IVS-II-1 (G > A) is the most frequent in Iran present in at least two thirds of the population. IVS-II-1 (G > A) has been observed from the north to the center and some parts of the south. Because of high rate of consanguinity marriage, the rate of IVS-II-1 (G > A) is high [24, 26].

**Region G**

IVS-I-5 (G > C) has been observed as most frequent mutation in the *β*-globin gene among the southern provinces, especially in southeastern provinces outlined as region G (Figure 1) [6, 28]. Its frequency reduces towards the center and disappears in the west and the north. Also, IVS-I-5 (G > C) is a frequent mutation in Arab countries, mostly in Eastern Asian Arab countries which may be explained by migration. This mutation known as the Asian/Indian mutation is very common in Indian subcontinent and Pakistan [29–31]. Detailed consideration revealed its frequency at its highest peak (90%) in the southern province of Hormozgan (region G-13, Figure 1) on the cost of the Persian Gulf. Yavarian et al. have reported 69.0% for IVS-I-5 (G > C) in this region [32]. Baysal reported that the IVS-I-5 (G > C) allele was introduced to the Arabian Peninsula by gene migration from Baluchistan, a region spanning Southern Iran, Afghanistan, and Pakistan [33]. This paper shows that gene migration from the province of Hormozgan (an economical center) has occurred over a long period during the past centuries [32, 34].

**Region H**

This area shows that IVS-II-1 (G > A) is the most frequent mutation followed by codons 8/9 (+G). The IVS-II-1 (G > A) allele is observed at variable frequencies in many countries. Chifu et al. declared multiple origins for this mutation [35]. The high frequency and multiple haplotypes indicate that the IVS-II-1 (G > A) mutation is native and one of the oldest *β*-Thalassemia defects in Iran.

The codons 8/9 (+G) mutation has been frequently observed after the IVS-II-1 (G > A) in most provinces of region H (Table 1), but there is exception like observed data in region H-18 (Table 1, Figure 1, Qazvin province) [36]. Also, the codon 30 (G > C) mutation has high frequency in regions H-16 and H-17 (Figure 1) in the north of Iran, which has appeared in lower frequency in other parts. It has been reported that the origin of codon 30 (G > C) mutation is related to United Arab Emirates [14], its frequency is about 3% in Lebanon and United Arab Emirates. Overall the codon 30 (G > C) mutation has frequency approximately 2% in Iran. Whereas, it seems there is no close relationship between the north of Iran and Arabian countries from population aspect. So we could propose another origin for codon 30 (G > C). Complementary studies showed a frequency of 17.5% for codons 36/37 (−T) in region H-12 [37], that could have relationship with two neighboring regions D-4 and D-5 which this mutation is frequent there. Hence codons 36/37 (−T) have been concentrated in the west toward center of Iran.

**Region I**

Finally, the codons 8/9 (+G) have been observed as the first *β*-globin mutation among the population of the north east with a frequency of approximately 65%, the highest frequency in comparison with other regions. The IVS-II-1 (G > A) and IVS-I-110 (G > A) mutations follow codons 8/9 (+G) with 10% and 8.4%, respectively. The codons 8/9 (+G) mutation is of Asian-Indian origin that the invasion and big migration of the Seljuk Turks and Mongol's invasions are the theories which support the transfer of codons 8/9 (+G) to the east and the northeast of Iran. The area indicated with an asterisk (Figure 1) represents Queshm Island, which has a sole mutation similar to that of the east Mediterranean origin. Codon 39 (C > T) is the most frequent mutation of *β*-Thalassemia among the western parts of the Mediterranean Sea such as Portugal, Spain, and France. This mutation is also the most frequent in Queshm Island, located south of Iran in the Persian Gulf. Portuguese colonial rule which lasted over a hundred years in the Persian Gulf may have been the main way for the entrance of this new gene into the gene pool of the population in this area [38].

## 4. Discussion

Iran has experienced admixture from various populations throughout history. Invasion and migration have important roles in genetic admixture. Basically, the Iranian people show heterogeneity, as reflected by other studies (unpublished data). The association studies between Turkey and the Arab countries in the East of the Mediterranean Sea have shown large number of *β*-Thalassemia mutations due to the heterogeneity of the population [32, 34, 39, 40]. We have not observed known mutations which are most frequent in all parts of Iran [9]. Recent studies have confirmed previous conclusions regarding the distribution of codons 8/9 (+G) in Iran [34]. Accordingly, It seems that codons 8/9 (+G) might reflect two circumstances; one connected to the secular trade along the great silk road which extends from Xian in China through the Indian subcontinent to Iran and the Eastern Mediterranean; the other to the invasion of the Mongols (1220 AD) and the Tatars (1380–87 AD), with respect to the prevalence of codons 8/9 (+G) in the Northeast, and toward center and northwest, it may be that the Turkish ethnicity may have affinities with the Oghuz Turk who migrated there from central Asia. The high occurrence of the codons 8/9 (+G) in Eastern Anatolia may also be the cause of its introduction to Turkey from Southern Asia [41]. 

Zahed reported that 52 mutations in the Arab world, as part of a study that included 13 countries representative of 21 Arabian nations [14], more than 47 observed mutations in the *β*-globin gene in Iran showed high heterogeneity among the population in comparison with Arabian people in area less than the Arab world. It is proposed that this heterogeneity has been derived originally from a population with an ethnic Aryan background; these results confirm earlier reports from other groups as well as could help to complete information of the population studies [7].

This paper also proposed new theories for the origin of 25 bp deletion, codons 36/37 (−T), and codon 30 (G > C) based on the recent investigations carried out in the southern and western and northern part of Iran, respectively (Regions D and E, Figure 1). It is clear that haplotypes determination could be useful in the case of analyzing the origin of hemoglobin gene, while it is often difficult to distinguish a new mutation or changes in haplotypes due to gene conversion. Up to now, there are not many studies regarding *β*-globin haplotypes in Iranian population only a few haplotypes analysis has been performed as published and unpublished data which the authors used for their conclusion. Some studies of the haplotypes background showed Iran known as the gate of Asia through which passed the important Silk Road could be attributed to a single and independent mutational event or due to the genetic admixture with other Asian populations like India, China, and Kuwait, the United Arab Emirates (UAE) and areas along the south shore of the Persian Gulf [42, 43]. 

Comparison of populations and haplotypes analyzed in the Mediterranean types revealed that the same polymorphism is associated with more population in the Iranian Thalassemic population than in the Mediterranean ones, suggesting that the Iranian Thalassemic population is more ancient than the Mediterranean one. On the other hand, the high heterogeneity and miscellaneous mutations among the Iranian population reflect the sociocultural background of this nation that due to a rich cultural education emphasizes fraternity and hospitality and urges population to accept newcomers with open arms. Also, this study can help the researchers doing low cost of test when they know which mutation has high rate in these regions.

## Figures and Tables

**Table 1:** Frequency of the *β*-thalassemia mutations among different provinces of the Iran*.

Provinces	Mutation															
	Sample size	IVS-II-1 (G > A)	IVS-I-5 (G > C)	Codons 8/9 (+G)	IVS-I-110 (G > A)	IVS-I-6 (T > C)	Codons 36/37 (−T)	25 bp del.	IVS-I-1 (G > A)	IVS-II-745 (G > C)	Codon 39 (C > T)	Codon 44 (−C)	Codon 30 (G > C)	Codon 5 (−CT)	Codon 8 (−AA)	Studied location
West Azerbaijan	75	**50.7%** ^ a^	8.0%	16.0%	5.3%	8.0%						9.3%	2.7%			A
East Azerbaijan	80	**32.0%**	2.6%	18.7%	16.0%				22.6%				5.3%			A
Ardebil	142	17.8%	7.0%	**30.0%**	25.4%	2.5%		0.6%	6.3%			6.3%		3.8%		A
Kurdistan	33	**31.8%**	1.5%	13.6%	1.5%	1.5%	1.5%					4.5%			6.0%	B
Khuzestan	208	12.0%	8.6%	17.3%	16.8%	10.5%			**23.3%**							C
Lorestan	65	27.7%	4.6%	10.7%	11.5%		**33.8%**	0.7%		1.6%		0.7%				D
Chaharmahal and Bakhtiari	57		29.8%		10.5%		**31.5%**		24.5%	3.5%		1.7%				D
Bushehr	104	13.0%	7.7%	8.2%	5.8%	1.5%		**24.4%**	9.1%	3.3%	3.3%		3.3%	2.4%		E
Fars	70	**47.1%**	1.4%		1.4%			21.4%		1.4%			1.4%		8.6%	F
Kerman	50	16%	**44%**	12.0%	4.0%											G
Sistan and Baluchistan	224	1.4%	**76.5%**	3.7%	11.1%			0.7%	0.7%		1.4%	0.7%		0.5%		G
Yazd	49	32.5%	**44.8%**	4.0%	6.1%				2.0%			2.0%				G
Hormozgan	70	4.2%	**90.0%**	5.8%												G
Esfahan	205	**29.0%**		26.0%	8.0%	2.9%	17.5%	2.0%			3.9%	4.4%			8.3%	H
Semnan	52	**40.3%**	5.7%	23.3%						3.8%			3.8%			H
Tehran	638	**42.4%**	8.4%	16.6%	7.8%	2.0%			7.5%	0.8%		0.1%	1.5%	1.3%		H
Mazandaran	77	**68.8%**								3.8%		1.3%	6.4%			H
Gilan	65	**49.2%**		10.7%			9.2%						9.2%	3.0%		H
Qazvin	60	**50.0%**	1.6%	5.0%	15.0%	3.3%						3.3%	3.3%		1.6%	H
Hamedan	47	**34.0%**	8.5%	19.1%	8.5%	2.1%			10.6%							H
Khorasan	70	10.0%		**65.7%**	8.5%											I

^a^It has been written in bold the most frequent mutations in each province.

*This table includes data from published data in local and international references and unpublished data during routinely diagnostic tests. Also the data is not available for blank areas.
